# Modern Sociology

**Published:** 1902-02-01

**Authors:** 


### MODERN SOCIOLOGY.

### SANITATION IN INDIA.

THE gradual spread of the plague over the greater part of the Indian Peninsula has brought vividly before the minds of most people the absence of all proper sanitation in the country generally; and the question at once arises, what are the authorities about to let such a state of things continue, and why, when plague does appear, do they not take measures to stamp it out at once, as was done in Liverpool and Glasgow?

In the first place it may be stated that it is not from want of knowledge that they do not take prompt action. The cold-weather visitor, shocked though he may be, sees little compared with the official who, in the burning winds of May and the sweltering heats of the rains, has to go in and out among the people, to leave the broader streets of the city, and plunge down the filthy lanes and gullies, to personally superintend the removal from a plague-stricken house of all the indescribable dirt that has collected in it for years, to live in daily and hourly intercourse with men who may have come straight from the bedside of a plague or small-pox patient, or even be sickening with disease themselves. He or his wife may daily inspect their own compound and the houses in it, but as often as not the servants go home to sleep, and custom, stronger than any law, requires them to visit the bazaar daily to buy their food. To forbid them to do so would mean that they would at once leave, and could not be replaced. An ayah will come straight from a child ill with small-pox to the children of her master, and in two cases personally known to me the unfortunate babies were only a week or two old and had not been vaccinated, and both died; the mothers fortunately escaped, though actually nursing the children. Now, as Government officials in India are well-educated English gentlemen, it is inconceivable that they should allow themselves and their families to run such risks daily unless they were positively forced to do so. But why are they?

Sanitation in England is of comparatively modern growth, as may be realised by reading not only descriptions of mediæval towns, but of the state of Old Edinburgh at the beginning of the nineteenth century, or even that of London at the time of the last cholera outbreak. Throughout the greater part of Europe now, however, the upper classes appreciate its importance, and with the spread of education the poorer are beginning to do so likewise. But in India, with few exceptions, neither rich nor poor, rajah nor peasant, have the least idea of the meaning of pure milk or water, of healthy dwellings and surroundings. And what is more, the very students and assistant doctors who pass good examinations in sanitary science will dwell contentedly under the most filthy conditions, such as no Englishman could endure for a week. It would be practically impossible for him, for instance, to take up his abode in the middle of Benares; the smells and noise alone would be enough to kill him. And yet well educated and wealthy natives actually prefer a house in a narrow lane, shut away from light and air, to one in the suburbs, and on account of the city's sanctity most Hindu princes maintain some sort of dwelling there to which they go at times for religious ceremonies and festivals. They simply do not seem to smell the smells which turn the Englishman sick, they eat food which would assuredly make him ill, and drink the milk of cows fed on all manner of trash, and kept in stables which are never properly cleaned out from one year's end to the other. For though the cow is sacred, it is not necessary to keep her clean, as every visitor to the Cow Temple at Benares knows. And they use literally any water for cleansing purposes, if not for drinking also. A woman will sit rubbing her brass cooking pots in a roadside puddle with a well within a few feet of her. I do not mean to say that they actually prefer dirty water to clean; the richer classes, indeed, will often send some distance to certain wells for their drinking water, but they do so solely on account of its superiority in flavour, never on sanitary grounds. Water, of course, can be made impure ceremonially, if, for instance, an Englishman accidentally touches the drinking vessel. But many orthodox Hindus hold that being a holy element it cannot convey illness, and so all theories of water-borne disease are of themselves impossible. The practical effects of this belief can be easily understood.

Further, few natives believe in infection, even by personal contact. If it is the will of God that you get ill, you will, in spite of all precautions; if it is not, you will escape. Infection by means of clothing is to them an unknown idea.

Fortunately there exists a strong belief in the efficacy of putting things out in the sun. When a native servant cleans a room, the first thing he does is to put everything out of doors, and as even in the cold weather the temperature rises to 130° or 140° in the sun, there is a very effective means of disinfection always at hand, and the blazing heats of May and June, when in the shade the temperature may not for weeks fall below 100°, effectively kill most germs. It has been consequently found that the water of the great rivers is singularly pure; the Jumna at Allahabad, for instance, after passing innumerable towns and villages, furnishes an exceptionally good analysis.

When such beliefs as stated above are reinforced by the innate conservatism of the East, the sanitary reformer's task is not an easy one. But most infectious diseases are dirt diseases, and here we come to one of his chief difficulties. None but the lowest and most degraded castes, whose very presence brings pollution to the orthodox Hindu, will have anything to do with human excreta. This is well-known to all Englishwomen in India, for an ayah of good caste will not even touch a child's napkin, and consequently the mother must either keep two attendants for her child, or else give it over to the care of one who is looked on by her fellow countrymen as the lowest of the low. It is true that Mahomedans should be free from these ideas, but in the greater part of India they are not, and I know a native Christian woman, Christian by birth and upbringing, who could not be trained as a sick nurse because she refused to give a bedpan to any but those of her own religion. These prejudices and their inevitable consequences undoubtedly account to a large extent for the filthy condition of the great Indian cities, and explain also the utter neglect by the previous rulers of the land of all matters of conservancy.

In a tropical country, moreover, it is exceedingly doubtful whether any system of underground drains is advisable, especially when the utter flatness of the plains is remembered. A leaking cesspool represents the native's greatest effort, and consequently the very soil in time gets poisoned, and must be abandoned. Ancient Delhi stretches nearly thirty miles down the river, and the former site of Benares, the high ground on either side of the Burna, a tributary of the Ganges, is quite uninhabited. An attempt was once made for strategical reasons to quarter some of the garrison there, but the men suffered too much in health.

(*To be continued.*)

